# Association between overweight/obesity and dental outcomes in early childhood: Findings from an Australian cohort study

**DOI:** 10.1111/cdoe.13006

**Published:** 2024-09-04

**Authors:** S. D. Leary, D. H. Ha, T. Dudding, L. G. Do

**Affiliations:** ^1^ Bristol Dental School University of Bristol Bristol UK; ^2^ School of Dentistry University of Queensland Brisbane Queensland Australia

**Keywords:** caries, gingivitis, longitudinal, obesity, periodontitis, plaque, preschool children

## Abstract

**Objectives:**

Oral health is an important part of general health and well‐being and shares risk factors, such as poor diet, with obesity. The published literature assessing the association between obesity and oral health in early childhood is sparse and inconsistent. The objective of this study was to investigate associations between overweight/obesity (measured by body mass index) and dental outcomes (caries, plaque index and gingival index) both cross‐sectionally and longitudinally, taking account of potential confounding factors, based on data collected at age 2 and age 5 within the Australian Study of Mothers' and Infants' Life Events Affecting Oral Health (SMILE) birth cohort study.

**Methods:**

This study used data from 1174 SMILE participants. Associations between overweight/obesity and dental outcomes were assessed using generalized linear regression models for the modified Poisson family with log link to estimate prevalence ratios. Cross‐sectional and longitudinal models were fitted, after minimal and full adjustment for potential confounders.

**Results:**

Approximately 12% of the participants were overweight/obese at 2 years and 9% at 5 years. Between 2 and 5 years, the prevalence of caries increased from approximately 4% to 24%, at least mild plaque accumulation increased from 37% to 90% and at least mild inflammation from 27% to 68%. There were no associations between overweight/obesity and the prevalence of dental caries; prevalence ratios (PR) [95% confidence interval (CI)] after adjustment for age and sex were 0.9 (0.3, 2.4) cross‐sectionally at 2 years, 1.0 (0.6, 1.5) cross‐sectionally at 5 years, and 1.0 (0.6, 1.5) for overweight/obesity at 2 years and caries at 5 years. Prevalence ratios were all around the value of 1 for the other dental outcomes and also after adjustment for additional confounders.

**Conclusions:**

There were no associations between overweight/obesity and dental caries, plaque index or gingival index in this cohort of preschool children. However, associations may emerge as the children become older, and it will be possible to extend analyses to include data collected at age 7 in the near future.

## 1 INTRODUCTION

Oral health is an important part of general health and well‐being, and oral diseases have serious health and economic burdens, greatly reducing the quality of life for those affected.^1^ Dental caries and periodontal disease are the most common oral health problems worldwide.^2^ Caries can begin in childhood, has been associated with undesired outcomes such as educational disadvantage^3^ and may require invasive treatment. Oral hygiene status, gingivitis and associated plaque accumulation in childhood have been associated with later development of dental caries, periodontal disease, subsequent tooth loss and also systemic diseases.^4^

Dental caries shares some risk factors with obesity such as the intake of free sugars and social deprivation, and it has been hypothesised that the two outcomes may co‐exist within the same individuals or populations.^5^, ^6^, ^7^ There are also many factors such as overall calorie intake and physical activity levels for obesity^8^ and exposure to fluoride and overall dietary composition for caries^9^ that are not directly associated with the other condition; those factors may be markers of common overall health behaviours that may be indirectly associated with the conditions. A number of studies have investigated childhood obesity as a potential risk factor for caries, and also, but to a lesser extent periodontal disease. Obesity has generally been measured by body mass index (BMI) and often combined with overweight. Caries has been measured by the number of decayed, missing or filled teeth (dmft), decayed, missing or filled surfaces (dmfs), decayed, extracted or filled surfaces (defs) or decayed or filled surfaces (dfs). Oral hygiene status has been measured by various indices including bleeding on probing (BOP), visual plaque index (VPI) or gingival bleeding index (GBI).

The most recent systematic review of obesity and dental caries in children aged 6 and younger was undertaken by Manohar et al.^10^ and based on nine studies. Six studies showed that children with overweight/obesity had more dental caries than those who were normal weight and the other three did not show any associations. As well as inconsistent findings, the quality of evidence varied considerably. The most recent systematic review of overweight/obesity and periodontal disease in children and adolescents was undertaken by Martens et al.^11^ and based on 12 studies. Although the meta‐analysis which used a subset of the studies showed an association between overweight/obesity and periodontal indices, only one study (not included in the meta‐analysis) was based on preschool children; Peng et al.^12^ had undertaken a case–control study on 324 five‐year‐olds in Hong Kong, and found that BMI was not associated with VPI. Another recent study systematically reviewed papers assessing the association between obesity and oral health in children and adolescents.^13^ Eighteen studies were included, but only five of those were preschool children, and all were from Mexico. Associations with overweight/obesity were inconsistent for caries, visible plaque and gingivitis.

The published literature on associations between obesity and both caries and periodontal disease in early childhood is therefore sparse, with inconsistent findings and variation in study quality. Well‐designed longitudinal studies with clinician‐measured dental outcomes and adjustments for potential confounders are essential. Hence, the aim of this study was to investigate associations between overweight/obesity (measured by BMI) and dental outcomes [caries, plaque index (PI) and gingival index (GI)] both cross‐sectionally and longitudinally, taking account of potential confounding factors, based on data collected at age 2 and age 5 within the Australian Study of Mothers' and Infants' Life Events Affecting Oral Health (SMILE) birth cohort study.

## 2 METHODS

### 2.1 Sample and study design

SMILE is a birth cohort study which has a long‐term goal of identifying and evaluating the relative importance and timing of critical factors that shape the oral health of young children. It is a single‐centre study conducted in Adelaide, Australia, with all newborns at the main three public hospitals between July 2013 and August 2014 (approximately 10 000) being eligible for inclusion; 2181 were enrolled. Participants were followed up with parent‐completed questionnaires when the child was 3 and 6 months and 1, 2 and 5 years old. Oral epidemiological examinations and anthropometric assessments were conducted at 2 and 5 years. Full details of the study can be found in the cohort profile paper.^14^ Ethics approval for SMILE was obtained from a number of Human Research Ethics Committees across South Australia (HREC#50.13,28/02/2013, HREC#13/WCHN/69,07/08/2013, HREC#H‐2018‐017,16/10/2018).

### 2.2 Questionnaires

Age‐specific questionnaires were sent to the parent/caregiver for all participants. Baseline questionnaires sent after recruitment at birth included information on the age of the mother at childbirth (grouped into ≤24 years, 25–34 years or 35+ years), mother's education completed (school, vocational, some university or higher), mother's country of birth (grouped into Australia/New Zealand/United Kingdom, India, other Asian country or other), household income (collected in 10 groups then combined into quartiles) and single‐parent status (yes or no). The Index of Relative Socio‐Economic Advantage and Disadvantage (IRSDA) was derived from reported postcodes.^14^ Pairs of deciles were combined, for example, deciles 1 and 2, to enable a five‐category variable to be used for analysis.

### 2.3 Physical assessment

The children underwent a physical assessment at ages 2 and 5 years. Calibrated electronic scales were used to measure weight (in kg) with light clothes. A medical standalone stadiometer was used to measure standing height (in cm) without shoes. The examination teams were trained to collect those measures in duplicate as recommended by the WHO Training Manual. Body mass index (BMI, kg/m^2^) was calculated using age‐ and sex‐specific BMI *Z*‐scores based on the WHO reference (WHO 1995).^15^ A *Z*‐score of less than −2 indicated underweight, −2 to +2 normal weight, +2 to +3 overweight and greater than +3 obese.

Detailed oral epidemiological examinations were undertaken, using criteria based on the existing standards for children.^16^ These included surface‐level assessment, visual–tactile assessment with the aid of compressed air, recording of stages of caries process (non‐cavitated, cavitated) and the use of differential diagnostic criteria. The examinations of children collected tooth surface‐level information on dental caries, and from this, dmft was derived; caries was defined as a dmft value greater than zero. Plaque samples were collected, and the plaque index was applied to indicate no, mild, moderate or abundant plaque.^17^ Additionally, a gingival index was applied to indicate no, mild, moderate or severe inflammation.^17^

### 2.4 Statistical methods

**FIGURE 1:**
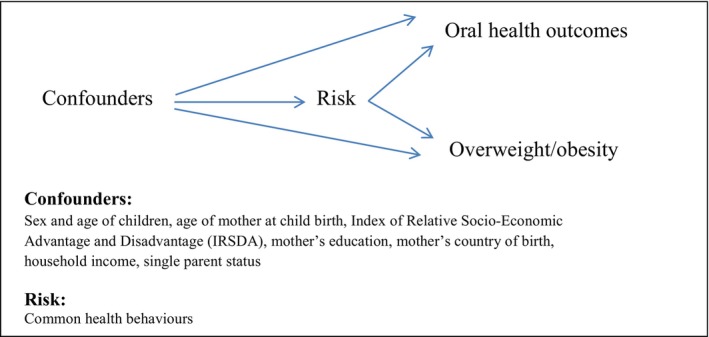
Graphical representation of the hypothetical framework for modelling.

All analyses were undertaken in Stata version 17 (StataCorp). Firstly, the characteristics of those who were included in this study were summarized using means/standard deviations (SDs) for continuous variables and frequencies/percentages for categorical variables. Next BMI, overweight/obesity, dmft/caries, PI and GI at both 2 and 5 years were summarized using means/SDs, medians/interquartile ranges (IQRs) or frequencies/percentages as appropriate. Associations between overweight/obesity (exposure) and the prevalence of dental outcomes (dmft >0, at least mild plaque, at least mild inflammation) were modelled using generalized linear regression models for the modified Poisson family with log link to estimate prevalence ratios. Two sets of cross‐sectional models (exposures and outcomes at age 2 or exposures and outcomes at age 5) and one set of longitudinal models (exposures at age 2, outcomes at age 5) were fitted. Potential confounders were selected using published literature, illustrated by the graphical representation of the hypothetical framework for modelling presented in Figure 1; minimally adjusted models included sex and age at exam/length of follow‐up as appropriate, and maximally adjusted models additionally included age of the mother at childbirth, IRSDA, mother's education, mothers' country of birth, household income and single parent status.

Planned sensitivity analyses included repeating the minimally adjusted models additionally adjusting for the relevant baseline dental measure (outcomes at 5 years only), restricting to those with data at both timepoints (cross‐sectional analyses only), and restricting to only those with full confounder information available to check whether changes after adjustment were due to missing data. As well as modelling caries prevalence, it was also possible to model caries severity by using the numerical dmft variable in generalized linear regression models for the negative binomial distribution with log link to estimate rate ratios, after minimal and full adjustment as above.

## 3 RESULTS

### 3.1 Sample

The numbers of children invited to attend the 2‐ and 5‐year examinations were 1988 and 1882, respectively. Of these, 1040 (52.3%) attended the 2‐year exam and 879 (46.7%) attended the 5‐year exam; this included 735 children who attended both exams. Analysis was based on the 1174 children who had both BMI and dental outcome data recorded for either the 2‐year exam only (307), 5‐year exam only (160) or both (707), that is, a total of 1014 for the 2‐year analysis and 867 for the 5‐year analysis.

Table 1 shows the characteristics of the 1174 children that were included in this analysis, of which 54% were male. The children were approximately 2.5 years and just under 5.5 years at the exams, while the average time between exams was just under 3 years.

**TABLE 1:** Characteristics of the 1174 children included in the analysis.

Characteristics	*N*	Mean (SD)
Age at 2‐year exam (years)	1014	2.5 (.3)
Age at 5‐year exam (years)	867	5.4 (.3)
Time between exams (years)	707	2.9 (.4)

Categories	*N*	Percentage
Child sex		
Male	631	53.8
Female	543	46.3
Age of mother at childbirth		
≤24 years	122	10.4
25–34 years	809	69.0
35+ years	242	20.6
IRSAD		
Deciles 1–2 (most disadvantaged)	201	17.3
Deciles 3–4	250	21.5
Deciles 5–6	230	19.8
Deciles 7–8	225	19.4
Deciles 9–10 (most advantaged)	257	22.1
Mother's education completed		
School	220	18.9
Vocational	295	25.4
Some university or higher	648	55.7
Mother's country of birth		
Australia, NZ, UK	854	73.1
India	99	8.5
Other Asian country	137	11.7
Other	78	6.7
Household income		
Q1 (lowest) ≤AU$40 k	160	13.7
Q2 AU41‐80 k	389	33.3
Q3 AU81‐120 k	353	30.2
Q4 (highest) AU121 k+	267	22.8
Single parent status		
Yes	67	5.8
No	1097	94.2

Abbreviations: BMI, body mass index; IRSAD, Index of Relative Socio‐economic Advantage and Disadvantage; NZ, New Zealand; Q, quartile; SD, standard deviation; UK, United Kingdom.

Body mass index and overweight/obesity plus the dental outcomes (dmft/caries, PI and GI) at both 2 and 5 years are summarized in Table 2. Approximately 12% were overweight/obese at 2 years and 9% at 5 years. The prevalence of caries increased from approximately 4% to 24%, at least mild plaque accumulation increased from 37% to 90% and at least mild inflammation from 27% to 68% between 2 and 5 years. Abundant plaque accumulation was only seen at 5 years (1.9%), and there was no severe inflammation at 2 or 5 years.

**TABLE 2:** Summary of BMI and dental outcomes.

Exposure/outcome	*N*	Mean (SD)
BMI *Z*‐score at 2 years	1014	.9 (1.1)
BMI *Z*‐score at 5 years	867	.6 (1.2)
	***N***	**Median (IQR)**
dmft if >0 at 2 years	39	2 (1, 2) (max = 16)
dmft if >0 at 5 years	206	2 (2, 2) (max = 20)

Categories	*N*	Percentage
OW/obese at 2 years		
Yes	119	11.7 (9.2% overweight/2.6% obese)
No	895	88.3
OW/obese at 5 years		
Yes	78	9.0 (5.8% overweight/3.2% obese)
No	789	91.0
Caries at 2 years		
Yes	39	3.9
No	975	96.1
Caries at 5 years		
Yes	206	23.8
No	661	76.2
Plaque index at 2 years		
At least mild plaque	379	37.4 (34.5% mild/2.9% mod)
No plaque	635	62.6
Plaque index at 5 years		
At least mild plaque	770	90.1 (60.1% mild/28.1% mod/1.9% abundant)
No plaque	85	9.9
Gingival index at 2 years		
At least mild inflammation	274	27.0 (23.6% mild/3.4% mod)
No inflammation	740	73.0
Gingival index at 5 years		
At least mild inflammation	579	67.7 (60.2% mild/7.5% mod)
No inflammation	276	32.3

Abbreviations: BMI, body mass index; dmft, decayed, missing or filled teeth; IQR, inter‐quartile range; OW, overweight; SD, standard deviation.

Associations between overweight/obesity and dental outcomes are shown in Table 3. All minimally adjusted prevalence ratios were very close to the null value of 1, with wide confidence intervals and large *p*‐values, so there was no clinical or statistical evidence of any associations either cross‐sectionally (exposures and outcomes both at 2 years or both at 5 years) or longitudinally (exposure at 2 years, outcomes at 5 years).

**TABLE 3:** Associations between overweight/obesity and dental outcome prevalence.

Coss‐sectional: age 2			Min. adjusted^a^ (max *N* = 1014)		Fully adjusted^b^ (max *N* = 994)	
	**Caries (2 year)**		**PR (95% CI)**	***p*‐value**	**PR (95% CI)**	***p*‐value**
	No	Yes				
**OW/Obese (2 year)**			0.9 (0.3, 2.4)	.8	0.9 (0.3, 2.5)	.9
No	860 (96.1%)	35 (3.9%)				
Yes	115 (96.6%)	4 (3.4%)				
	**Plaque (2 year)**					
	None	At least mild				
**OW/Obese (2 year)**			0.9 (0.7, 1.1)	.3	0.9 (0.7, 1.1)	.3
No	556 (62.1%)	339 (37.9%)				
Yes	79 (66.4%)	40 (33.6%)				
	**Gingivitis (2 year)**					
	None	At least mild				
**OW/Obese (2 year)**			1.0 (0.7, 1.3)	.96	1.0 (0.7, 1.4)	.9
No	654 (73.1%)	241 (26.9%)				
Yes	86 (72.3%)	33 (27.7%)				

Cross‐sectional: age 5			Min. adjusted^a^ (max *N* = 867)		Fully adjusted^b^ (max *N* = 848)	
	**Caries (5 year)**		**PR (95% CI)**	***p*‐value**	**PR (95% CI)**	***p*‐value**
	No	Yes				
**OW/Obese (5 year)**			1.0 (.6, 1.5)	.8	.9 (.6, 1.4)	.8
No	601 (76.2%)	188 (23.8%)				
Yes	60 (76.9%)	18 (23.1%)				
	**Plaque (5 year)**					
	None	At least mild				
**OW/Obese (5 year)**			1.0 (1.0, 1.1)	.3	1.0 (1.0, 1.1)	.4
No	80 (10.3%)	699 (89.7%)				
Yes	5 (6.6%)	71 (93.4%)				
	**Gingivitis (5 year)**					
	None	At least mild				
**OW/Obese (5 year)**			1.0 (.8, 1.1)	.7	1.0 (.8, 1.1)	.6
No	251 (32.2%)	528 (67.8%)				
Yes	25 (32.9%)	51 (67.1%)				

Longitudinal			Min. adjusted^a^ (max *N* = 707)		Fully adjusted^b^ (max *N* = 692)	
	**Caries (5 year)**		**PR (95% CI)**	***p‐*value**	**PR (95% CI)**	***p*‐value**
	No	Yes				
**OW/Obese (2 year)**			1.0 (.6, 1.5)	.8	1.0 (.6, 1.5)	.9
No	480 (76.6%)	147 (23.4%)				
Yes	62 (77.5%)	18 (22.5%)				
	**Plaque (5 year)**					
	None	At least mild				
**OW/Obese (2 year)**			1.0 (1.0, 1.1)	.1	1.0 (1.0, 1.1)	.1
No	61 (9.8%)	559 (90.2%)				
Yes	4 (5.1%)	74 (94.9%)				
	**Gingivitis (5 year)**					
	None	At least mild				
**OW/Obese (2 year)**			1.1 (1.0, 1.3)	.2	1.1 (.9, 1.3)	.2
No	215 (34.7%)	405 (65.3%)				
Yes	21 (26.9%)	57 (73.1%)				

Abbreviations: CI, confidence interval; OW, overweight; PR, prevalence ratio.

^a^ Minimally adjusted for sex and age at exam (for cross‐sectional analyses)/length of follow‐up (for longitudinal analyses).

^b^ Additionally adjusted for age of mother at childbirth, Index of Relative Socio‐Economic Advantage and Disadvantage (IRSDA), mother's education, mother's country of birth, household income, single‐parent status.

Prevalence ratios were almost identical if further adjustment was made for confounders (age of mother at childbirth, IRSDA, mother's education, mother's country of birth, household income, single parent status; see final column of Table 3). Table S1 shows the minimally adjusted models with additional adjustments for baseline dental measures for 5‐year outcomes, and also restricting the cross‐sectional analyses to those with data available at both timepoints; again findings were similar, although confidence intervals were wider due to smaller numbers being included in the analyses. As there were very little missing confounder data, it was not deemed necessary to repeat minimally adjusted models restricted to those with full confounder information.

Associations between overweight/obesity and caries severity are shown in Table 4. All rate ratios, minimally and fully adjusted, were close to 1, with large *p*‐values, so there was no clinical or statistical evidence of any associations either cross‐sectionally (exposures and outcomes both at 2 years or both at 5 years) or longitudinally (exposure at 2 years, outcomes at 5 years).

**TABLE 4:** Associations between overweight/obesity and caries severity.

	*N*	Min. adjusted^a^		*N*	Fully adjusted^b^	
		RR (95% CI)	*p*‐value		RR (95% CI)	*p‐*value
Cross‐sectional: age 2	1014	0.7 (0.2, 2.1)	.5	994	0.7 (0.2, 2.2)	.6
Cross‐sectional: age 5	867	1.4 (0.8, 2.6)	.2	848	1.4 (0.8, 2.4)	.2
Longitudinal	707	1.0 (0.5, 1.7)	.9	692	1.1 (0.6, 2.0)	.7

Abbreviations: CI, confidence interval; OW, overweight; RR, rate ratio.

^a^ Minimally adjusted for sex and age at exam (for cross‐sectional analyses)/length of follow‐up (for longitudinal analyses).

^b^ Additionally adjusted for age of mother at childbirth, Index of Relative Socio‐Economic Advantage and Disadvantage (IRSDA), mother's education, mother's country of birth, household income, single‐parent status.

## 4 DISCUSSION

No associations were found between overweight/obesity and clinician measured dental outcomes in early childhood. These findings were based on a large contemporary cohort of Australian children and used robust statistical methods including longitudinal as well as cross‐sectional modelling, adjustment for potential confounders and investigation of caries severity as well as prevalence.

Limitations of this study include the measurements used and the representativeness of the analysis sample. Body mass index is a measure of body size that is only a proxy for body fat, so it may have been preferable to use an alternative measure such as fat mass derived from dual x‐ray absorptiometry.^18^ However, using BMI instead of fat mass in the current study allowed a fairer comparison of findings with the literature, and clinician‐measured rather than self‐reported height and weight measurements were used to increase accuracy.^19^ Dental caries was measured by dmft recorded by trained examiners. However, when the within‐child 2‐ and 5‐year data were compared, it appeared that 1.2% of the caries improved over time. Caries reversal due to examiner misclassification is a well‐known problem in longitudinal research,^20^ and further work will investigate methods for adjusting caries increments for reversals. The PI has some limitations, primarily it is subjectivity, but despite this, it is widely used in epidemiological studies.^21^ The GI has been criticized for the difference between scoring no or mild inflammation being subjectively based on visual assessment, but it is generally suggested that the index is sensitive and reproducible.^21^

It is possible that the findings of this study would have been different if all those originally recruited were able to be included in the analysis sample. As shown by Do et al.^14^ the attrition rate from the SMILE baseline/birth sample of 2181 was higher than expected and higher among those women with lower SES. However, attempts had been made to over‐sample people from lower socio‐economic areas at baseline, to ensure that the socio‐economic profile reflected that of South Australian mothers as far as possible, despite the attrition. In addition, the original sampling frame of the three hospitals covered 67% of all births in Adelaide,^22^ and the IRSAD distribution is fairly even across the five pairs of deciles (17%–22%), which is based on information from the 5‐yearly Australian Census of Populations and Housing.^23^ Although there was some missing confounder information, less than 2% of the sample were omitted in the fully adjusted models, and restricting the cross‐sectional analyses to those with data available at both timepoints gave very similar results compared to using all available data. The prevalence of overweight/obesity in the current study was approximately 12% at 2 years and 9% at 5 years, so lower than has been reported previously in Australian children,^24^ for example, the prevalence of overweight/obesity in a 2003 study of 4‐year‐old South Australian children was approximately 20%,^25^ likely due to the socio‐economic profile of those remaining in the SMILE study. The prevalence of caries in the current study rose to 24% by age 5, similar to the prevalence of 25.3% (95% CI 20.5, 30.8) for 5–6‐year olds in South Australia based on data from the National Child Oral Health Study 2012–2014.^26^

The published literature includes a mixture of cross‐sectional and longitudinal studies on this topic, none of which were undertaken in Australia. The results from the current study support the findings from three of the studies in the Manohar et al.^10^ review of overweight/obesity and caries,^27^, ^28^, ^29^ the study from the Martens et al.^11^ review with VPI as the outcome,^12^ and two of the studies from the Aceves‐Martins et al.^13^ review with caries as the outcome.^30^, ^31^ In contrast to the current study findings, the Manohar et al.^10^ review identified six studies which demonstrated associations between overweight/obesity and caries. Possible reasons for inconsistent funding include different study designs,^32^, ^33^, ^34^, ^35^ and older ages,^36^, ^37^ as well as different locations (Sweden, Canada, USA, Brazil, Chile, India). Some variation in findings between studies may be due to the differing economic status between settings, as evidenced in a review by Hayden et al.^38^ The Aceves‐Martins et al.^13^ review identified three studies that showed associations with either caries^39^ or periodontal measurements,^40^, ^41^ but all used different indices to the current study and were undertaken in Mexico.

Findings from the current study are not surprising given the inconsistency of the literature. It might be that associations between overweight/obesity and dental outcomes emerge as the children become older and caries prevalence/incidence increase; 7‐year data have been collected for the SMILE cohort and are currently being cleaned which will then allow extended analyses to include this third timepoint. In addition to using BMI at age 2 as the exposure for later dental outcomes, all repeated measures of BMI data can be used to derive trajectories which can also be used as exposures. Ideally, further data sweeps of the SMILE children will be undertaken, to allow longer follow‐up and therefore exploration of the long‐term associations between overweight/obesity and dental outcomes. Identifying associations will suggest that weight loss interventions to improve oral health should be considered, although it will be important to investigate the potential mediating effects of variables such as free sugar intake.^42^ At the other end of the spectrum, associations between underweight and dental outcomes have been emerging, for example, these associations are shown in the Public Health England report on approximately 67 000 UK 5 year olds.^43^ While it is not possible to replicate this analysis within SMILE as there are so few underweight children (0.7% at 2 years and 2.0% at 5 years), other studies with larger numbers of children in this category should investigate associations with dental outcomes.

In conclusion, no associations between overweight/obesity and dental caries, plaque index and gingival index have been demonstrated in this cohort of preschool children. There is a need for confirmation of these findings in other well‐designed cohorts of preschool children with repeated measurements of dental outcomes and confounder information available. It is possible that associations may emerge as children get older, and the analyses presented in this paper will be repeated when 7‐year data are available.

## AUTHOR CONTRIBUTIONS

SD Leary selected and performed the appropriate statistical analyses, wrote the first draft and produced updated drafts of the manuscript. DH Ha collected and prepared the dataset and commented on drafts of the manuscript. T Dudding commented on drafts of the manuscript. LG Do developed the concept, ran the study, and commented on drafts of the manuscript.

## FUNDING INFORMATION

The SMILE birth cohort is funded by Australian National Health and Medical Research Council Project Grants #APP1046219 2013‐17 and APP144595 2018‐22.

## CONFLICT OF INTEREST STATEMENT

None.

## PATIENT CONSENT STATEMENT

Parents of SMILE participants provided written informed consent at the baseline and at subsequent physical assessments.

## Supporting information


Table S1.

## Data Availability

Data may be available from the researchers for reasonable requests and with appropriate ethical considerations.
