# Supplementary material for: Association between overweight/obesity and dental outcomes in early childhood: Findings from an Australian cohort study
Source: Community Dent Oral Epidemiol. 2024 Sep 4;53(1):50–7. doi: 10.1111/cdoe.13006 (PMC11754151; doi:10.1111/cdoe.13006)
Supplement: Supplementary file 1 — Table S1. [file CDOE-53-50-s001.docx]

**Supplementary Table 1**: Associations between overweight/obesity and dental outcome prevalence; sensitivity analyses

| **CROSS-SECTIONAL: AGE 2** | **Outcome** |  | **Min. adjusted* including relevant baseline dental measure** | |  | **Min. adjusted* restricting to those with data at both timepoints (Max N=707)** | |  |
| --- | --- | --- | --- | --- | --- | --- | --- | --- |
|  |  |  | **PR (95% CI)** | **P value** | | **PR (95% CI)** | **P value** | |
| OW/Obese (2y) | Caries (2y) |  | N/A | N/A | | 1.5 (0.5, 4.1) | 0.5 | |
|  |  |  |  |  | |  |  | |
| OW/Obese (2y) | Plaque (2y) |  | N/A | N/A | | 0.8 (0.6, 1.2) | 0.3 | |
|  |  |  |  |  | |  |  | |
| OW/Obese (2y) | Gingivitis (2y) |  | N/A | N/A | | 1.1 (0.8, 1.6) | 0.5 | |
|  |  |  |  |  | |  |  | |
| **CROSS-SECTIONAL: AGE 5** |  |  | **(Max N=723)** | |  | **(Max N=707)** | |  |
|  |  |  | **PR (95% CI)** | **P value** | | **PR (95% CI)** | **P value** | |
| OW/Obese (5y) | Caries (5y) |  | 1.0 (0.6, 1.6) | 0.9 | | 0.9 (0.6, 1.5) | 0.8 | |
|  |  |  |  |  | |  |  | |
| OW/Obese (5y) | Plaque (5y) |  | 1.0 (1.0, 1.1) | 0.4 | | 1.0 (1.0, 1.1) | 0.2 | |
|  |  |  |  |  | |  |  | |
| OW/Obese (5y) | Gingivitis (5y) |  | 1.0 (0.8, 1.2) | 0.98 | | 1.0 (0.9, 1.2) | 0.8 | |
|  |  |  |  |  | |  |  | |
| **LONGITUDINAL** |  |  | **(Max N=707)** | |  |  | |  |
|  |  |  | **PR (95% CI)** | **P value** | | **PR (95% CI)** | **P value** | |
| OW/Obese (2y) | Caries (5y) |  | 0.9 (0.6, 1.4) | 0.7 | | N/A | N/A | |
|  |  |  |  |  | |  |  | |
| OW/Obese (2y) | Plaque (5y) |  | 1.0 (1.0, 1.1) | 0.1 | | N/A | N/A | |
|  |  |  |  |  | |  |  | |
| OW/Obese (2y) | Gingivitis (5y) |  | 1.1 (1.0, 1.3) | 0.2 | | N/A | N/A | |
|  |  |  |  |  | |  |  | |

OW = overweight; PR = prevalence ratio; CI = confidence interval

*minimally adjusted for sex and age at exam (for cross sectional analyses)/length of follow-up (for longitudinal analyses)
